# Evaluation of the peritumoral features using radiomics and deep learning technology in non-spiculated and noncalcified masses of the breast on mammography

**DOI:** 10.3389/fonc.2022.1026552

**Published:** 2022-11-21

**Authors:** Fei Guo, Qiyang Li, Fei Gao, Chencui Huang, Fandong Zhang, Jingxu Xu, Ye Xu, Yuanzhou Li, Jianghong Sun, Li Jiang

**Affiliations:** ^1^ Department of Radiology, Harbin Medical University Cancer Hospital, Harbin, Heilongjiang, China; ^2^ Deepwise Artificial Intelligence Lab, Beijing Deepwise and League of PHD Technology Co., Ltd, Beijing, China; ^3^ Department of Oncology, Harbin Medical University Cancer Hospital, Harbin, Heilongjiang, China

**Keywords:** non-spiculated and noncalcified masses, mammography, peritumoral features, deep learning, radiomics

## Abstract

**Objective:**

To assess the significance of peritumoral features based on deep learning in classifying non-spiculated and noncalcified masses (NSNCM) on mammography.

**Methods:**

We retrospectively screened the digital mammography data of 2254 patients who underwent surgery for breast lesions in Harbin Medical University Cancer Hospital from January to December 2018. Deep learning and radiomics models were constructed. The classification efficacy in ROI and patient levels of AUC, accuracy, sensitivity, and specificity were compared. Stratified analysis was conducted to analyze the influence of primary factors on the AUC of the deep learning model. The image filter and CAM were used to visualize the radiomics and depth features.

**Results:**

For 1298 included patients, 771 (59.4%) were benign, and 527 (40.6%) were malignant. The best model was the deep learning combined model (2 mm), in which the AUC was 0.884 (P < 0.05); especially the AUC of breast composition B reached 0.941. All the deep learning models were superior to the radiomics models (P < 0.05), and the class activation map (CAM) showed a high expression of signals around the tumor of the deep learning model. The deep learning model achieved higher AUC for large size, age >60 years, and breast composition type B (P < 0.05).

**Conclusion:**

Combining the tumoral and peritumoral features resulted in better identification of malignant NSNCM on mammography, and the performance of the deep learning model exceeded the radiomics model. Age, tumor size, and the breast composition type are essential for diagnosis.

## Introduction

Breast cancer is a type of disease with high heterogeneity, and its incidence has been increasing in many countries worldwide (1). In low- and middle-income countries of Asia, breast cancer has become a significant public health concern (2). As one of the most effective breast cancer screening tools (3, 4), mammography can reduce the mortality rate (5, 6). Breast mass is one of the main signs that can be detected on mammography, and differential diagnosis between benign and malignant lesions is the focus of radiologists’ work (7). In practice, proper preoperative breast mass evaluation can better assist clinicians in making treatment decisions.

On mammography, spiculated masses are one of the apparent signs of malignancy (8, 9). If calcifications accompany the mass, additional information on the differential diagnosis may be provided based on the types of calcifications (10, 11). However, non-spiculated and noncalcified masses (NSNCM) are more commonly detected on mammography. The mammographic differential diagnosis is mainly based on morphology in clinical practice. For instance, a circumscribed margin usually indicates a benign mass. However, some malignant tumors present as circumscribed masses on mammography (12, 13). Accurate diagnosis of breast mass on mammography are still a challenge, especially in early diagnosis of breast cancer (14).

Radiomics transforms medical images into high-dimensional data through high-throughput quantitative feature extraction (15), which is gaining importance in cancer research (16). Furthermore, the imaging features can be automatically extracted using deep learning technology to replace manually designed features (17). For instance, studies on the classification of masses on mammography have been reported, including tumors with a spiculated margin or calcification (18–22). However, mass imaging analysis only focuses on the characteristics of the tumor itself (23–25).

Breast cancer consists of neoplastic cells and significant alterations in the surrounding stroma or tumor microenvironment (26, 27). Tumors and the surrounding area can be said to consist of spatially organized ecosystems, wherein tumor cells and the immune contextures of the different compartments are in dynamic interplay with potential clinical effect (28). These histological changes are reflected in medical images to varying degrees (29, 30). Few studies have focused on the peritumoral features and classified mammographic NSNCMs, although some papers have concerned classification on ultrasound and magnetic resonance imaging (31, 32). The objective of the present study was to evaluate the peritumoral features based on radiomics and deep learning for the classification of NSNCM on mammography.

## Materials and methods

### Patients

This retrospective analysis was approved by the Research Ethics Committee of Harbin Medical University Cancer Hospital (approval # KY2021-04). Due to its retrospective and anonymous characteristics, required informed consent from each participant was waived.

We retrospectively screened the digital mammography data of 2254 patients who underwent surgery for breast lesions in Harbin Medical University Cancer Hospital from January to December 2018. Potential subjects with any of the following were excluded: spiculated masses with or without calcification, or non-spiculated masses with calcification; history of local mastectomy or neoadjuvant therapy; breast biopsy before examination; multiple masses; phyllodes tumor; fat-containing masses; and masses near chest wall not fully included or blurred mammograms due to inability to tolerate pressure. The endpoint of interest was the postoperative pathological results of the lesion, categorized as benign or malignant. For all included patients, their pathological types were recorded.

### Data acquisition, segmentation, and peritumoral region

The mammograms of included patients were obtained with a full-field digital mammography system (MS-3500, Fuji, Japan; Inspiration, Siemens, Germany). In the vast majority of cases, images were acquired through the automatic exposure mode of the device. But a few cases cannot be pressurized because the mass is large or hard, experienced physicians used manual exposure to obtain images. Conventional craniocaudal and mediolateral oblique views were obtained. The examinational pressure was based on the patient’s maximum tolerance.

Two radiologists manually segmented the tumoral region of interest (ROI), and the segmentation tool used for annotation was Deepwise Multi-modal Research Platform V1.0. The attending radiologist had 8 years’ experience. For a mass with a density different from the surrounding parenchyma, the ROI was carefully delineated along the margin of the mass. If a mass had no density difference with the surrounding parenchyma but with a halo, the ROI was carefully delineated along with the inner halo. If there was no density difference and no halo sign, the margin was delineated as far as possible based on the convex contour of mass. The tumoral ROI was reviewed and corrected by an associate chief radiologist with 16 years’ mammographic experience. Both two radiologists reached a consensus after discussion for inconsistent cases.

**Figure 1:**
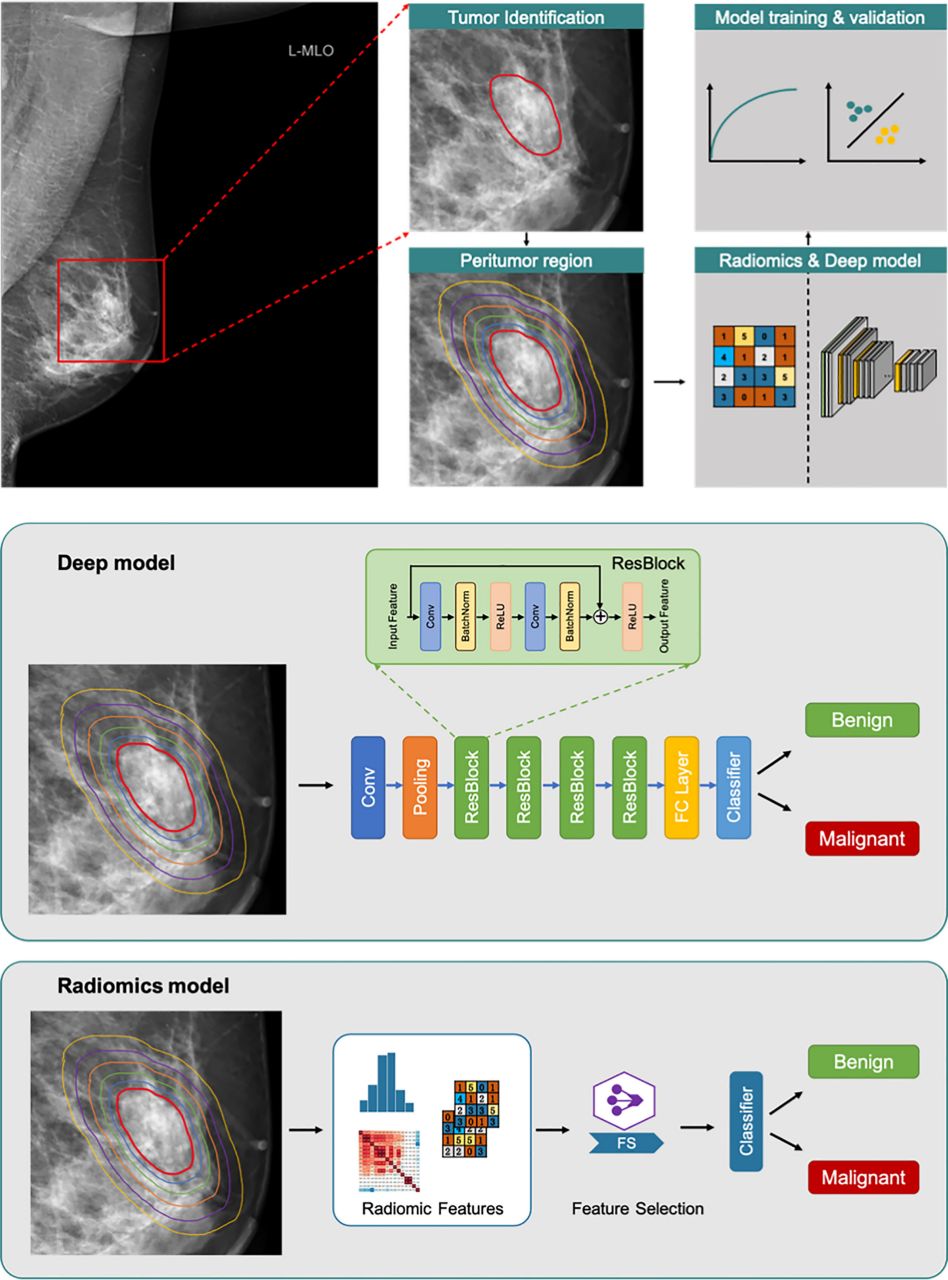
Overall flowchart of the method. Tumor region (red line). Peritumoral regions (lines with different colors outside red line). The radiomics and deep models respectively for benign/malignant prediction.

Subsequently, the peritumoral region was automatically generated. Firstly, the dilation processing of the morphological image operation was used to expand the contour of the tumoral region at a different range. Then, the peritumoral and combined regions were obtained by subtracting and maintaining the original tumoral region, respectively. Finally, the region of the breast and pectoral muscles were automatically extracted by the Otsu threshold method. Regions other than the breast and pectoral muscles in the peritumoral range were removed to obtain the final peritumoral region. Finally, 1 mm, 2 mm, 3 mm, 4 mm, and 5 mm were expanded (**Figure 1**).

### Deep learning and radiomics models

**Figure 2:**
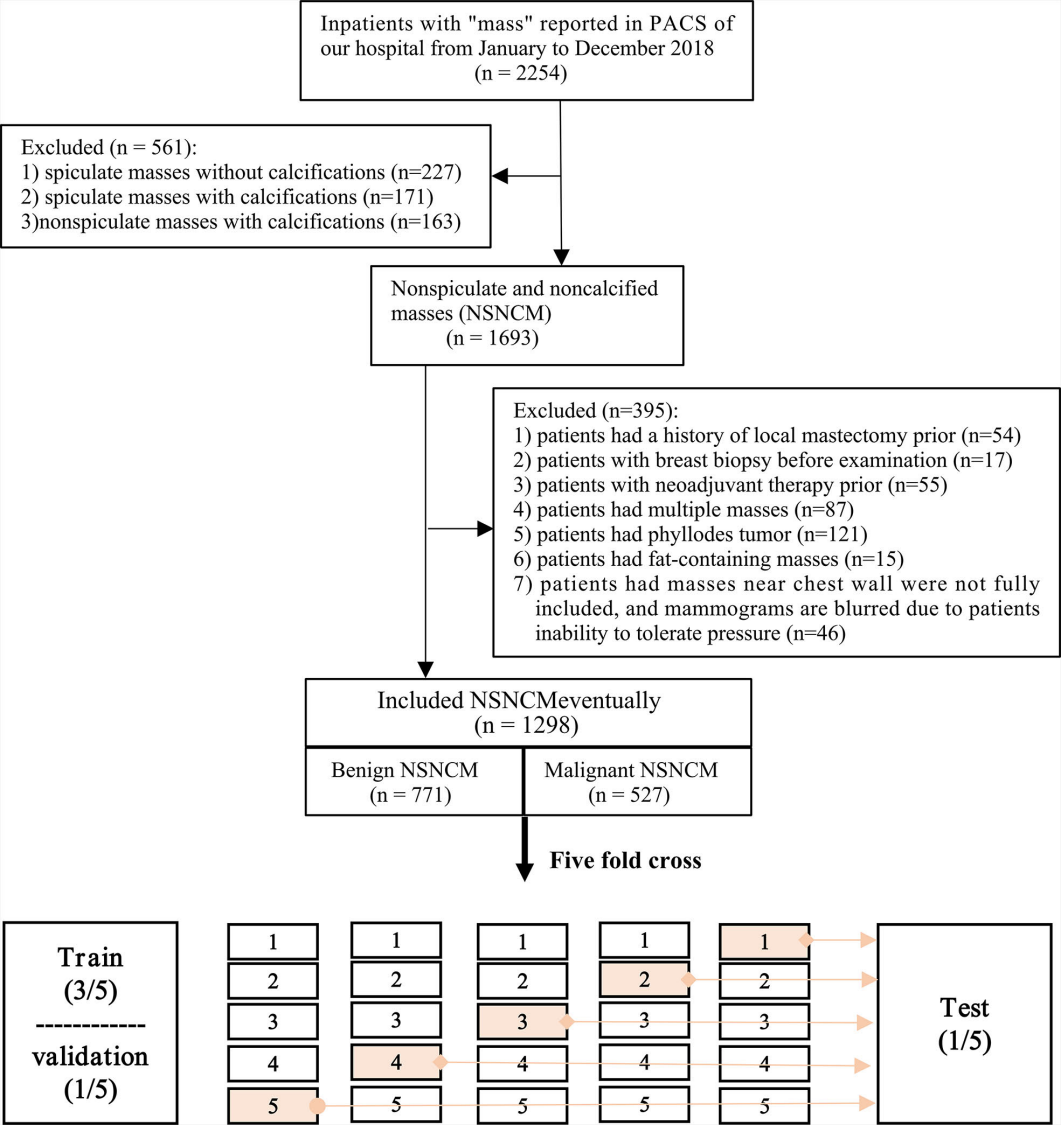
Flow chart of inclusion and exclusion of patients and division of data set.

The flow chart of the classification is shown in **Figure 1**. Residual Network-based deep learning classifiers and logistic regression-based radiomics classifiers (30-34) were separately used to distinguish benign and malignant NSNCM. In the model building, all cases were randomly divided into five sets with equal proportions for five-fold cross-validation. We divided the training set, the validation set and the test set in a 3:1:1 ratio (**Figure 2**). In each fold, the five equivalent sets were taken as test set successively, resulting in five test sets. The model with the highest AUC was selected from the validation set as the final model, and the final evaluation result of the model is the mean value of the 5 test sets. The mammographic features were extracted from 3 types of regions (i.e., tumoral region, peritumoral regions with different distances, and combined regions, including tumoral and peritumoral regions) to train and validate the models, respectively.

Before fed into the ResNet model, each input mass image was resized to 256 × 256 and normalized to mean value of zero and standard deviation of one. In the training phase, data augmentation methods of horizontally and vertically flipping the image were employed. The ResNet structure adopted in this study was the standard ResNet-34. We used a pre-trained weights on about 1.3 million natural images of 1000 object classes from the ImageNet database. The final classification layer (1000-way softmax) of the pre-trained ResNet-34 was replaced with a single output with sigmoid operation that predict the malignant probability of the input mass image. The loss function was binary cross-entropy loss. All the layers of the ResNet model were optimized using an initial learning rate of 0.001 with a weight decay rate of 0.00001. The learning rate was reduced by a factor of 0.1 after the validation loss plateaued for ten epochs. The optimization was performed by the stochastic gradient descent (SGD) optimizer with the momentum of 0.9. The training epoch was set as 100 and the batch size was 64. We implemented the networks using the open-source PyTorch (https://pytorch.org/) deep learning framework. We used four Nvidia Titan Xp GPUs for training acceleration. The training time of each batch was 0.4s and the total training time of one fold was about 30 minutes.

To interpret how the deep learning model automatically discriminates suspicious malignant lesions in the mammogram, we used the class activation map (CAM) to visualize the model features (33).

In the radiomics model, we used 6 calculation methods provided by the open-source image toolbox Pyradiomics to extract radiomics features (34). These radiomics calculations consisted of the following: first-order statistics; shape (2-dimensional); gray-level co-occurrence matrix (GLCM); gray level run length matrix (GLRLM); gray level size zone matrix (GLSZM); and gray level dependence matrix (GLDM). A total of 825 radiomics features were extracted from each region of interest. To avoid model overfitting, the joint hypothesis test was used for feature selection. When the linear correlation coefficient between any 2 features was more significant than a threshold (0.9), the one with less influence on the benign/malignant classification was removed. Next, a logistic regression algorithm was employed to construct a classification model, and L1 regularization was introduced to mitigate overfitting further. Finally, we used sensitivity, specificity, and receiver operating characteristic (ROC) curves to assess the performance of different models, and model analyses were performed at both the ROI level and the patient level.

### Clinical characteristics analysis

Patients in each set were divided into 4 groups based on age in years as follows: ≤20, 20–40,40–60, >60. Two radiologists respectively reviewed the mammographic views on the specialized diagnostic workstation without knowledge of the pathological diagnosis. Breast compositions were evaluated and recorded using BI-RADS lexicon (35), including entirely fatty (type A); scattered areas of fibroglandular density (type B); heterogeneously dense (type C); and extremely dense (type D). The mass sizes were recorded and based on largest diameter were analyzed as either ≤20 mm or >20 mm.

### Statistical analysis

Patient and tumor characteristics were compared with two-tailed two-sample t test for continuous variables and chi-square cross-tabulation for categorical variables. In all experiments, five-fold cross validation was performed for model evaluation. The performance of all the models were evaluated by using the area under the receiver operating characteristic curve (AUC). The statistical significance for the difference among models performance in AUC was assessed by the DeLong test using dedicated in-house software written in python 3.6.10. All statistical tests were two-sided, and P < 0.05 indicated significant.

## Results

### Participants

Overall, we screened 2254 patients while adhering to the inclusion and exclusion criteria (**Figure 2**). Eventually, 1298 patients with NSNCM were included in the study, aged 45.7 ± 11.8 years. Among the included patients, the NSNCM of 771 and 527, respectively, were benign and malignant. Among the benign masses, there were 348 (254) cases of fibroadenoma (adenosis); 107 (36) cases of ductal papilloma (inflammation); and 25 (1) cases of cyst (tubular adenoma). Among the malignant masses, there were 439 (61) cases of non-special (particular) invasive ductal carcinoma; and 23 (4) cases of ductal carcinoma *in situ* (lymphoma).

There were significant differences in age, tumor size, and breast composition type between the benign and malignant groups (P < 0.001; **Table 1**). Specifically, the patients in the benign group were mainly between 20 and 60 years old, while those in the malignant group were mainly over 40 years old. Benign tumors were mostly ≤20 mm, while most malignant NSNCM were larger. The major breast composition type of patients in the benign and malignant groups was type C.

**Table 1:** Clinical characteristics between benign and malignant NSNCM, n (%).

	Total	Benign	Malignant	χ2	P ^a^
Subjects, n	1298	771	527		
Age, y				252.6	<0.001
≤20	18 (1.4)	18 (2.3)	Nil		
20-40	414 (31.9)	348 (45.1)	66 (12.5)		
40-60	715 (55.1)	383 (49.7)	332 (63.0)		
>60	151 (11.6)	22 (2.9)	129 (24.5)		
Size, mm				61.701	<0.001
≤20	682 (52.5)	475 (61.6)	207 (39.3)		
>20	616 (47.5)	296 (38.4)	320 (60.7)		
Breast composition type ^b^				61.399	<0.001
A	32 (2.5)	9 (1.2)	23 (4.4)		
B	111 (8.6)	37 (4.8)	74 (14.0)		
C	1083 (83.4)	666 (86.4)	417 (79.1)		
D	72 (5.5)	59 (7.6)	13 (2.5)		

a Chi-square test;

b Breast composition types: A, entirely fatty; B, scattered areas of fibroglandular density; C, heterogeneously dense; D, extremely dense.

### Performance of radiomics models in different regions

**Figure 3:**
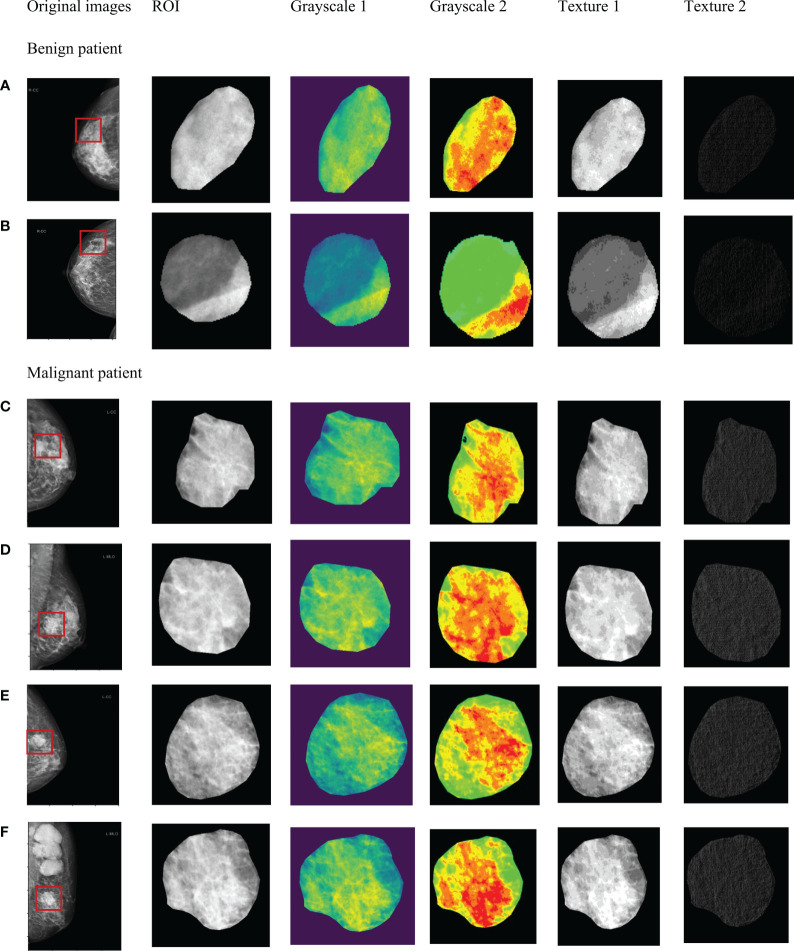
Significant radiomics features of masses in tumor ROI. Benign masses **(A, B)**. Malignant masses **(C–F)**.

After correlation analysis and L1 regularization feature selection, the initial 825 radiomics features of each ROI significantly reduced redundant features. Finally, the modeling features of the three radiomics models were respectively as: tumoral features (n=88, tumoral radiomics model), peritumoral features (n=107, peritumoral radiomics model), combined features (n=130, combined radiomics model), which were summarized in **Table S1** (Supplementary material). As shown in **Figure 3**, the internal texture features of malignant tumors were complex, showing pixel gray and denser structure (red in **Figure 3**).

For the prediction of all lesions, the AUC results using features within different regions (tumoral, peritumoral, combined) to predict benign and malignant NSNCM are summarized in **Table 2**. At the patient level, the AUC of the model with the tumoral region feature is 0.774. Regarding the peritumor models, all the performances are comparable with the tumoral model (P > 0.05). As for the models with combined region, significant performances are achieved at a peritumoral distance of 1 mm (AUC = 0.786, P = 0.046) and 2 mm (AUC = 0.793, P = 0.012), while others (3-5 mm) get comparable results. The best performance is reached at a peritumoral distance of 2 mm, for both the peritumor and combined models. The AUCs at the ROI level suggest similar results in **Table 3**.

**Table 2:** Benign/malignant classification performance of radiomics models in patient level.

	Size, mm	AUC (95% CI)	Sensitivity	Specificity	P *
Tumoral		0.774 [0.748, 0.799]	0.732 (386/527)	0.678 (523/771)	Reference
Peritumoral	1	0.763 [0.737, 0.789]	0.725 (382/527)	0.660 (509/771)	0.276
	2	0.774 [0.749, 0.800]	0.713 (376/527)	0.686 (529/771)	0.968
	3	0.771 [0.745, 0.796]	0.713 (376/527)	0.684 (527/771)	0.748
	4	0.772 [0.747, 0.798]	0.719 (379/527)	0.691 (533/771)	0.896
	5	0.765 [0.739, 0.791]	0.708 (373/527)	0.676 (521/771)	0.365
Combined	1	0.786 [0.761, 0.811]	0.729 (384/527)	0.702 (541/771)	0.046
	2	0.793 [0.768, 0.817]	0.772 (407/527)	0.673 (519/771)	0.012
	3	0.775 [0.750, 0.801]	0.784 (413/527)	0.619 (477/771)	0.857
	4	0.780 [0.754, 0.805]	0.740 (390/527)	0.669 (516/771)	0.437
	5	0.770 [0.744, 0.795]	0.736 (388/527)	0.668 (515/771)	0.579

* Compared with tumoral.

CI, confidence interval.

**Table 3:** Benign/malignant classification performance of radiomics models in ROI level.

	Size, mm	AUC (95% CI)	Sensitivity	Specificity	P *
Tumoral		0.761 [0.744,0.778]	0.722 (780/1081)	0.653 (1457/2231)	Ref.
Peritumoral	1	0.749 [0.732,0.766]	0.708 (765/1081)	0.654 (1458/2231)	0.116
	2	0.762 [0.745,0.779]	0.727 (786/1081)	0.670 (1494/2231)	0.847
	3	0.760 [0.743,0.777]	0.740 (800/1081)	0.645 (1438/2231)	0.899
	4	0.759 [0.742,0.776]	0.692 (748/1081)	0.674 (1504/2231)	0.818
	5	0.753 [0.736,0.770]	0.728 (787/1081)	0.634 (1415/2231)	0.317
Combined	1	0.775 [0.759,0.792]	0.721 (779/1081)	0.683 (1524/2231)	0.001
	2	0.778 [0.762,0.795]	0.737 (797/1081)	0.682 (1521/2231)	0.002
	3	0.770 [0.753,0.787]	0.765 (827/1081)	0.632 (1409/2231)	0.079
	4	0.776 [0.759,0.792]	0.734 (793/1081)	0.666 (1485/2231)	0.009
	5	0.767 [0.750,0.784]	0.721 (779/1081)	0.684 (1527/2231)	0.269

* Compared with tumoral.

CI, confidence interval.

### Performance of deep learning models in different regions

The benign/malignant classification performance of the pre-trained network for all lesions under different conditions is summarized in **Table 4**. At the patient level, the model with the tumoral region achieved an AUC of 0.838. The performance of model with a peritumoral distance of 2 mm (AUC = 0.861) was significantly better than the tumoral model (P = 0.033). The performance of the models with a peritumoral distance of 3 to 5 mm were comparable to that of the tumoral model. The performances of all the models with combined regions were significantly better than that of the tumoral model. The best performance is also reached at a peritumoral distance of 2 mm for combined models (AUC = 0.884, P = 0.001), as in the radiomics models. Similar comparison results are also presented at the ROI level (**Table 5**).

**Table 4:** Benign/malignant classification performance of deep learning models in patient level.

	Size, mm	AUC (95% CI)	Sensitivity	Specificity	P *
Tumoral		0.838 [0.815,0.861]	0.753 (397/527)	0.741 (571/771)	
Peritumoral	1	0.805 [0.780,0.829]	0.744 (392/527)	0.721 (556/771)	0.005
	2	0.861 [0.840,0.882]	0.776 (409/527)	0.796 (614/771)	0.033
	3	0.849 [0.827,0.871]	0.812 (428/527)	0.738 (569/771)	0.22
	4	0.834 [0.811,0.856]	0.763 (402/527)	0.764 (589/771)	0.738
	5	0.847 [0.825,0.869]	0.769 (405/527)	0.759 (585/771)	0.28
Combined	1	0.865 [0.844,0.886]	0.808 (426/527)	0.789 (608/771)	0.003
	2	0.884 [0.863,0.904]	0.820 (432/527)	0.817 (630/771)	0.001
	3	0.860 [0.838,0.881]	0.801 (422/527)	0.780 (601/771)	0.006
	4	0.872 [0.852,0.893]	0.810 (427/527)	0.785 (605/771)	0.001
	5	0.863 [0.842,0.884]	0.786 (414/527)	0.803 (619/771)	0.005

* Compared with tumoral.

CI, confidence interval.

**Table 5:** Benign/malignant classification performance of deep learning models in ROI level.

	Size, mm	AUC (95% CI)	Sensitivity	Specificity	P *
Tumoral		0.826 [0.810,0.841]	0.763 (825/1081)	0.708 (1580/2231)	
Peritumoral	1	0.793 [0.776,0.809]	0.741 (801/1081)	0.731 (1630/2231)	<0.001
	2	0.844 [0.830,0.859]	0.747 (808/1081)	0.788 (1759/2231)	0.018
	3	0.831 [0.816,0.846]	0.753 (814/1081)	0.748 (1668/2231)	0.464
	4	0.825 [0.810,0.841]	0.751 (812/1081)	0.757 (1689/2231)	0.974
	5	0.830 [0.815,0.846]	0.743 (803/1081)	0.756 (1687/2231)	0.519
Combined	1	0.851 [0.836,0.865]	0.825 (892/1081)	0.741 (1653/2231)	0.027
	2	0.870 [0.857,0.884]	0.800 (865/1081)	0.797 (1779/2231)	<0.001
	3	0.849 [0.834,0.864]	0.791 (855/1081)	0.767 (1711/2231)	0.025
	4	0.849 [0.834,0.864]	0.781 (844/1081)	0.774 (1726/2231)	0.002
	5	0.852 [0.837,0.866]	0.796 (860/1081)	0.765 (1707/2231)	0.021

* Compared with tumoral.

CI, confidence interval.

The CAM analysis showed that our model after training was responsive to the suspicious mass areas that exhibited changes, which the radiologists also identified. The model feature assessments of the CAM results are presented in **Figure 4**. The deep features extracted from tumoral, peritumoral distances of 1,2,3,4,5mm were shown respectively, representing the highlighted activation area in the input image. In the activation map, activation degree is denoted as continuous pixel values, and in brief, red, yellow, and blue represent high, medium, and low activation, respectively. In **Figure 4-1**, CAM status of tumoral and peritumoral regions were observed on 8 patients with malignant masses, all the regions were correctly predicted. While those of 6 patients with malignant masses partially predicted correct were shown in **Figure 4-2**, tumoral region predictions were wrong, and proximal peritumoral region predictions as follows:1) regions of both 1 mm and 2 mm were correct. 2) regions of 1 mm were wrong, regions of 2 mm were correct. 3) regions of both 1 mm and 2 mm were wrong.

**Figure 4-1:**
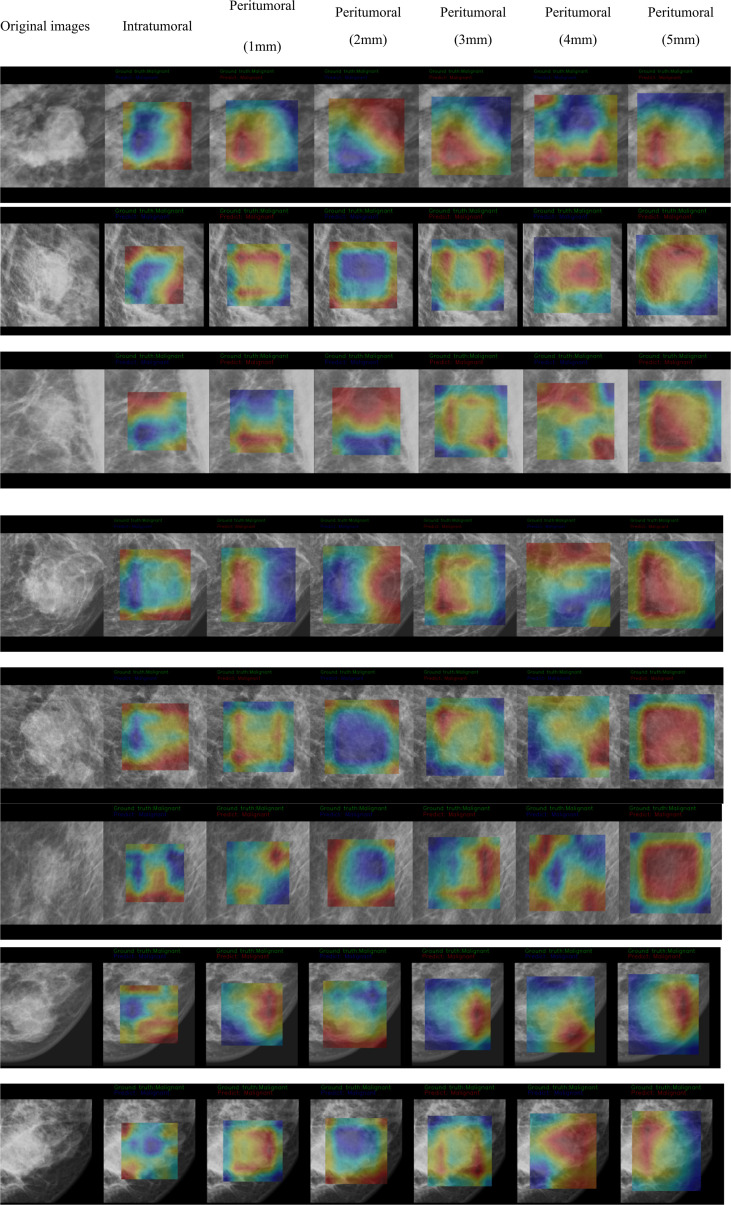
CAM status of tumoral and peritumoral regions were observed on 8 patients with malignant masses. All the regions were correctly predicted.

**Figure 4-2:**
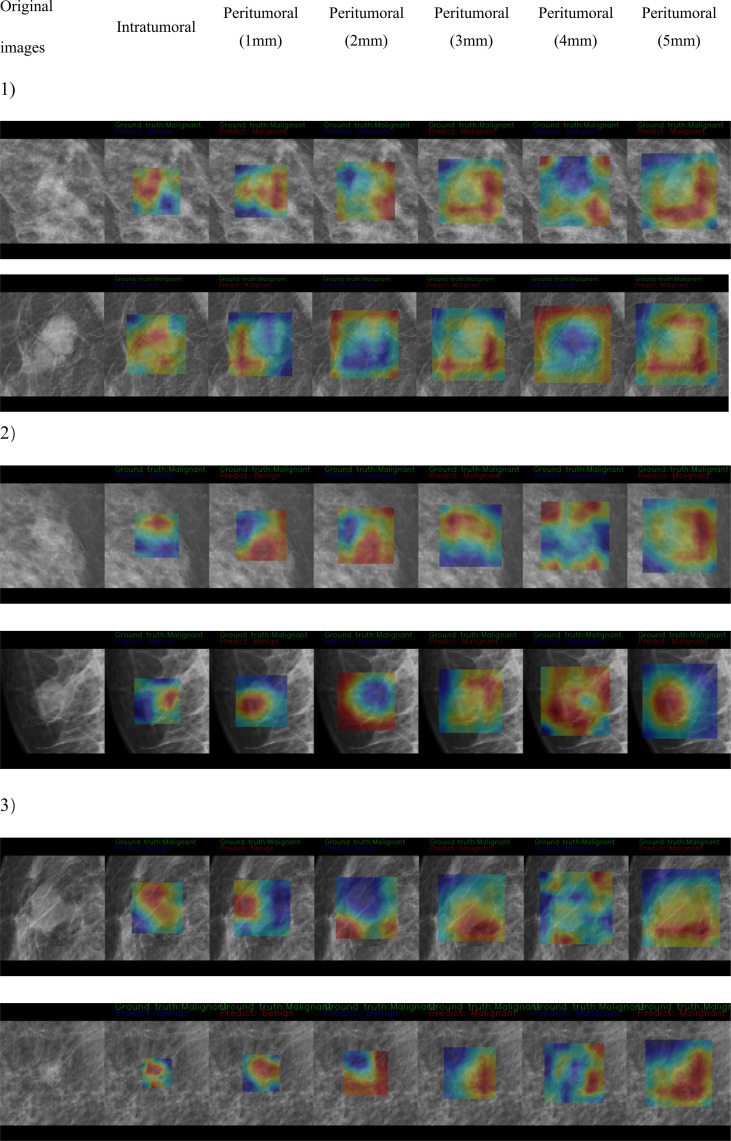
CAM status of tumoral and peritumoral regions were observed on 6 patients with malignant masses. Tumoral region predictions were wrong. Proximal peritumoral region predictions as follows:1) regions of both 1 mm and 2 mm were correct. 2) regions of 1 mm were wrong, regions of 2 mm were correct. 3) regions of both 1 mm and 2 mm were wrong.

**Figure 5:**
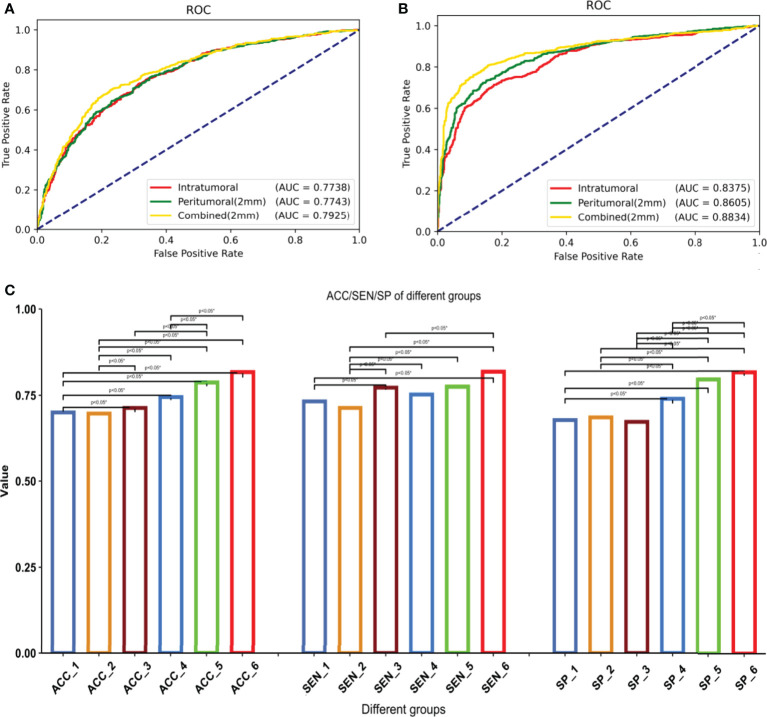
1–6 are respectively: radiomics-tumoral; radiomics-peritumoral 2 mm; radiomics-combined 2 mm; deep learning-tumoral; deep learning-peritumoral 2 mm; deep learning-combined 2 mm models. **(A)** ROC curve and AUC of the radiomics models, including 1–3. **(B)** ROC curve and AUC of the deep learning models, including 4-6. **(C)** Differences among all models in accuracy, sensitivity, and specificity. *Statistical significance.

The models with the highest AUC under the two methods of radiomics and deep learning were selected for detailed comparison. We showed the ROC curves/AUC of the six models, and the significance test of ACC, SEN and SPE among different models in **Figure 5**. The ROC curve showed that the deep learning combined regions (2mm) model had the highest AUC and were significantly better than all the other models (P < 0.05). All the deep learning models were significantly better than the models based on radiomics (P < 0.05). In the radiomics models, the combined regions (2mm) model were significantly better than the tumoral model (P < 0.05), while there was no significant difference between the other radiomics models (P > 0.05). When considering ACC, SEN and SPE, all these indicators of deep learning combined regions (2mm) model were the highest, and were significantly higher than those of all other deep learning and radiomics models (P < 0.05) except for deep learning peritumoral (2mm) (P > 0.05).

### Influence of age, tumor size, and breast composition type

**Figure 6:**
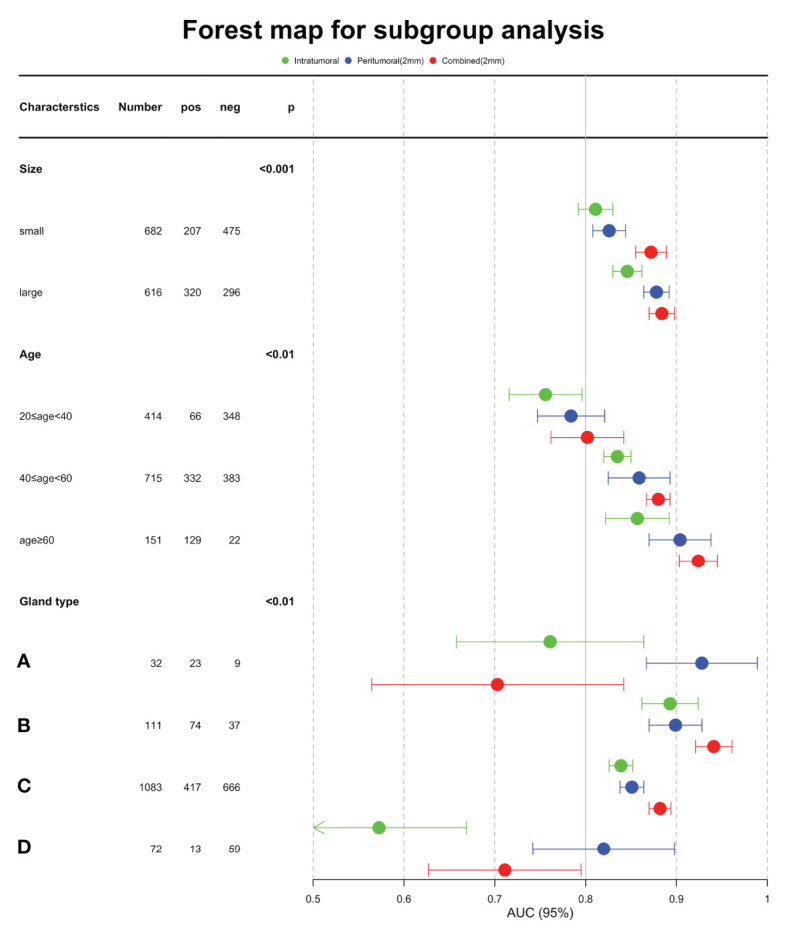
Forest map displayed the AUC of the three models in subgroup analysis.

**Table 6** showed the AUC performance of the three deep learning models in different tumor sizes, patient ages and breast composition types. The results showed that the AUC of the large mass (diameter > 20mm) group was significantly higher than that of the small mass (diameter ≤ 20mm) group (P < 0.05), and the age > 60 group was significantly higher than that of 40 < age ≤ 60 and 20 < age ≤ 40 groups (P < 0.05). The AUCs of the combined model in all the sizes and ages groups was higher than or comparable with tumoral and peritumoral models. In different breast composition types, the AUCs of group B was significantly higher than other three types (P < 0.05) and group C followed in both tumoral and combined models (P < 0.05). While in peritumoral models, group A and B showed higher performance and then the group C and D followed. From the aspect of models, we noticed that the combined models showed higher performance than tumoral and peritumoral models in group B and C (P < 0.05), especially AUC of breast composition type B which reached 0.941, while in the groups A and D the peritumoral models were better than the other models (P < 0.05). Forest map displayed the AUC of the three models in subgroup analysis, and it is clear that the combined model was better (**Figure 6**).

**Table 6:** AUC of three regions (Tumoral, Peritumoral of 2mm, Combined of 2 mm) in subgroups (Size, Age, breast composition types) ^a^.

	Group	Total	Positive	Negative	Tumoral	Peritumoral ^b^	Combined ^b^
Size, mm	Small	682	207	475	0.811 ± 0.019	0.826 ± 0.018	0.872 ± 0.017
	Large	616	320	296	0.846 ± 0.016	0.878 ± 0.014	0.884 ± 0.014
Age, y	20 to 40	414	66	348	0.756 ± 0.040	0.784 ± 0.037	0.802 ± 0.040
	40 to 60	715	332	383	0.835 ± 0.015	0.859 ± 0.034	0.880 ± 0.013
	>60	151	129	22	0.857 ± 0.035	0.904 ± 0.034	0.924 ± 0.021
Composition	A	32	23	9	0.761 ± 0.103	0.928 ± 0.061	0.703 ± 0.139
type	B	111	74	37	0.893 ± 0.031	0.899 ± 0.029	0.941 ± 0.020
	C	1083	417	666	0.839 ± 0.013	0.851 ± 0.013	0.882 ± 0.012
	D	72	13	59	0.572 ± 0.097	0.820 ± 0.078	0.711 ± 0.084

a Reported as n, unless indicated otherwise

b 2 mm.

## Discussion

In this study, we developed deep learning and radiomics models that combined the tumoral and peritumoral image features of masses to explore the high correlation factors on mammography for effectively distinguishing benign and malignant NSNCM. Three regions, including tumoral, peritumoral distances of 1, 2, 3, 4, 5 mm, and corresponding combined regions, were used to construct Resnet-based deep learning classifiers and LR-based radiomics classifiers. The classification performance of the combined model was superior to that of the peritumoral and tumoral models, whether based on deep learning or radiomics. In addition, some improvement of the peritumoral model relative to the tumoral was shown by the deep learning method. This confirmed that peritumoral features contributed greatly to identify malignant NSNCM on mammography. The best model was the deep learning combined with peritumoral 2 mm region, which had high diagnostic performance. This indicated that the peritumoral region was noteworthy before making clinical treatment decisions.

We applied deep learning models in the study, and performance exceeded that of the radiomics models. The significantly different models in deep learning may be caused by the different feature extraction methods between radiomics and deep learning. In deep learning models, with hierarchical convolutional layers and down-sampling effect, extracted features fused more information at different scales. When the data is sufficient, the deep learning model mines richer features, so the performance is usually better. Therefore, the overall high diagnostic performance of deep learning may potentially assist patient management.

While, there were some limitations. Firstly, the data set came from a single center, which may influence its external validity. Therefore, we planned a multi-center analysis in the future to further verify our conclusions. About the tumoral ROI, both two radiologists reached a consensus after discussion for inconsistent cases. The intraclass correlation coefficients was not carried out. Secondly, we did not establish a quantitative or qualitative mapping relationship between the malignant probability value of artificial intelligence and the BI-RADS evaluation classification criteria. The mapping relationship can help apply the artificial intelligence model in clinical practice to assist radiologists in diagnosis and to assist radiologists in understanding the artificial intelligence model better. Finally, adding more factors, such as breast density (36), clinical features (37), and semantic features (38), may help improve the diagnostic performance. In the future, we will explore these factors in depth.

However, some practical implications were found in this study. Combining tumoral and peritumoral features helped distinguish benign from malignant masses. Some studies that focused on tumoral masses used machine learning to construct the classifier for benign and malignant masses, to achieve an accuracy of over 85% on mammography (25, 39, 40). In addition, Yan et al. (39) found that combining imaging biomarkers improved the prediction of benign and malignant breast masses.

Our study designed various ROI extraction strategies containing mass and context features, with 3 main results. Firstly, the classification ability of the combined model was significantly higher than that of the pure tumoral or peritumoral model. The rationale may fall into the following mechanisms: desmoplastic reaction (41), increased lymphedema, and extracellular matrix remodeling in the peritumoral area (42) may be markers of local malignancy, which were considered to be a response of the host tissue against tumor (43). Yi et al. (44) similarly found that peritumoral regions showed abnormal ADC values.

Secondly, the 2mm peritumoral region might provide more information to distinguish between benign and malignant masses, while the 1mm and 3,4,5mm peritumor regions were mediocre and comparable with each other. This suggests indirectly that malignant tumor cell invasion is more likely to occur actively in the peritumoral distance of 2 mm. Shin et al. (45) presumed that there might be more active tumor proliferation proximal to the tumor.

Thirdly, the performance of deep learning models exceeds that of the radiomics models. The results showed that the classification capability of the deep learning model with combined region of 2mm was significantly higher than that of all the other models. In the radiomics model, the AUC of the combined model was significantly higher than that of the pure tumoral and peritumoral models. After a detailed analysis of the radiomics features, we found that GLCM, GLSZM, and first-order features all had high weights in the 3 regional models (tumor, peritumor, and combination), showing that the texture and grayscale information may be more valuable in differentiating benign and malignant masses.

Based on the statistical analysis, we reported the relative influence of the subgroups on ability of the deep learning model to classify. The chi-squared test showed significant differences between the benign and malignant groups in size, age, and breast composition type (P < 0.001). We further explored the hierarchical analysis results of the deep learning model, which showed that the model achieved significantly higher AUC for large size, age older than 60 years, and breast composition type B. This suggests that it is essential to improve further diagnosis for groups categorized as small mass, other age, and breast composition types. Risk stratification with clinical characteristics is necessary.

## Conclusion

Combining the tumoral and peritumoral features could best identify malignant NSNCM on mammography. The performance of the deep learning model exceeded the radiomics model. In addition, age, tumor size, and breast composition type are essential for the diagnosis. These findings can contribute to patient management before clinical treatment decisions, and provide research ideas in NSNCM.

## Data availability statement

The datasets presented in this article are not readily available because of privacy. Requests to access the datasets should be directed to JS, jianghong713@sina.cn.

## Ethics statement

The studies involving human participants were reviewed and approved by Research Ethics Committee of Harbin Medical University Cancer Hospital (approval # KY2021-04). Written informed consent for participation was not required for this study in accordance with the national legislation and the institutional requirements.

## Author contributions

JS, LJ, FGu, FGa, and FZ: The paper design; JS, FGu, QL, FGa, and FZ: Manuscript writing; JS, LJ, FGu, QL, YX, and YL: Data acquisition and literature retrieval; FGa, FZ, CH, and JX: Software technical support, literature retrieval and statistics. All authors critically reviewed the report, and approved the submitted version.

## Funding

The subject was supported by the Natural Science Foundation of Heilongjiang Province (LH2019H101).

## Conflict of interest

Authors FGa, CH, FZ, and JX are employed by Beijing Deepwise & League of PHD Technology Co., Ltd.

The remaining authors declare that the research was conducted in the absence of any commercial or financial relationships that could be construed as a potential conflict of interest.

## Publisher’s note

All claims expressed in this article are solely those of the authors and do not necessarily represent those of their affiliated organizations, or those of the publisher, the editors and the reviewers. Any product that may be evaluated in this article, or claim that may be made by its manufacturer, is not guaranteed or endorsed by the publisher.

## Abbreviations

AUC, area under the receiver operating characteristic curve; BI-RADS, Breast Imaging Reporting and Data System; CAM, class activation map; GLCM, gray-level co-occurrence matrix; GLDM, gray-level dependence matrix; GLRLM, gray-level run-length matrix; GLSZM, gray-level size zone matrix; NSNCM, non-spiculated and noncalcified masses; ROC, receiver operating characteristic; ROI, region of interest.
