# Supplementary material for: Evaluation of the peritumoral features using radiomics and deep learning technology in non-spiculated and noncalcified masses of the breast on mammography
Source: Front Oncol. 2022 Nov 21;12:1026552. doi: 10.3389/fonc.2022.1026552 (PMC9721450; doi:10.3389/fonc.2022.1026552)
Supplement: Supplementary file 1 [file DataSheet_1.pdf]

Supplementary Material

Table S1: Radiomics features and coefficients involved in modeling

(Tumoral, Peritumoral, Combined)

1）88 Tumoral radiomics features and coefficients

feature	coef
wavelet-HHL_glcm_JointEntropy	0.6348
wavelet-LLH_glszm_ZonePercentage	-0.4505
wavelet-LHL_firstorder_RootMeanSquared	-0.4139
wavelet-LLH_firstorder_Skewness	0.411
original_glcm_Imc1	0.39
wavelet-LLH_firstorder_Variance	0.3731
original_glcm_DifferenceAverage	0.352
wavelet-LLH_firstorder_Range	-0.3062
wavelet-LLH_glcm_SumEntropy	0.2943
wavelet-LHH_glszm_GrayLevelNonUniformity	0.2321
original_glcm_DifferenceVariance	0.2235
wavelet-LHH_glcm_Contrast	0.2112
wavelet-HHL_glcm_Correlation	-0.2026
wavelet-HHL_glcm_Id	-0.1956
wavelet-LHH_firstorder_Kurtosis	-0.1856
original_firstorder_Maximum	-0.1739
wavelet-LHH_glcm_SumSquares	-0.166
wavelet-LLH_glrlm_ShortRunHighGrayLevelEmphasis	0.1645
wavelet-LLL_firstorder_Kurtosis	0.1543
wavelet-HHH_firstorder_Minimum	0.1524
wavelet-LLH_firstorder_RootMeanSquared	0.1485
wavelet-LHH_glrlm_RunLengthNonUniformityNormalized	-0.1426
wavelet-LHL_glcm_Correlation	-0.1399
wavelet-HLH_glcm_SumSquares	-0.1379
wavelet-HHH_glcm_ClusterProminence	0.1336
wavelet-HLH_glrlm_GrayLevelVariance	-0.1322
wavelet-LLL_glcm_InverseVariance	-0.1244
original_firstorder_Range	-0.1108
wavelet-HLH_gldm_SmallDependenceLowGrayLevelEmphasis	0.1093
wavelet-HHL_firstorder_Skewness	0.1083
wavelet-HHH_firstorder_Mean	0.1059
wavelet-LLH_firstorder_10Percentile	-0.1011
original_glcm_Imc2	0.0987

wavelet-LLH_glszm_ZoneEntropy	-0.0977
wavelet-LLL_firstorder_Skewness	-0.0976
wavelet-LHH_glszm_SizeZoneNonUniformityNormalized	-0.0944
wavelet-LHH_firstorder_Range	0.0933
wavelet-HHL_firstorder_Median	0.0932
original_glszm_SmallAreaEmphasis	0.0873
wavelet-HLL_firstorder_Minimum	0.0824
wavelet-LLH_glrlm_LongRunLowGrayLevelEmphasis	-0.0817
wavelet-HLH_glrlm_LongRunLowGrayLevelEmphasis	0.0769
wavelet-HLH_glcm_Idm	-0.0727
wavelet-HLL_glcm_SumSquares	0.0722
wavelet-HLL_glcm_Idmn	-0.0709
wavelet-HHH_glszm_SizeZoneNonUniformity	-0.0705
wavelet-HHH_gldm_DependenceVariance	0.0693
wavelet-HHH_glrlm_ShortRunHighGrayLevelEmphasis	-0.0669
wavelet-HLL_firstorder_Mean	0.0662
original_shape_Elongation	0.059
wavelet-HLH_firstorder_Kurtosis	-0.0577
wavelet-HLH_glcm_Autocorrelation	-0.0548
wavelet-HLH_firstorder_Minimum	-0.0534
wavelet-HLH_glszm_GrayLevelNonUniformityNormalized	0.0489
wavelet-LHH_glrlm_ShortRunLowGrayLevelEmphasis	-0.0465
wavelet-HLH_glcm_Imc2	-0.0443
wavelet-HLL_glszm_LargeAreaEmphasis	-0.0441
wavelet-HHH_glszm_ZoneEntropy	-0.0417
wavelet-LHH_firstorder_10Percentile	-0.0416
wavelet-HHH_glszm_SmallAreaHighGrayLevelEmphasis	0.0412
wavelet-HLH_glszm_GrayLevelNonUniformity	0.0385
wavelet-LHH_glszm_ZoneEntropy	0.0375
wavelet-HLL_glcm_Correlation	-0.0355
wavelet-HLH_glszm_SmallAreaHighGrayLevelEmphasis	-0.0348
wavelet-HHH_firstorder_Kurtosis	0.0331
wavelet-HLH_firstorder_Skewness	-0.0321
wavelet-LHH_firstorder_Maximum	0.0311
wavelet-LHL_glcm_Id	-0.0307
wavelet-HHH_glcm_SumEntropy	0.0283
wavelet-HHH_glszm_SizeZoneNonUniformityNormalized	0.0241
wavelet-LHL_firstorder_Median	-0.0232
wavelet-LHH_glszm_SmallAreaLowGrayLevelEmphasis	-0.0226
wavelet-LHL_firstorder_Minimum	-0.0206

wavelet-LHH_gldm_SmallDependenceHighGrayLevelEmphasis	-0.0186
wavelet-HHL_glcm_ClusterShade	-0.0173
wavelet-HLL_firstorder_Skewness	-0.0165
wavelet-HLH_glszm_SizeZoneNonUniformityNormalized	-0.0113
wavelet-HHL_glcm_ClusterProminence	-0.0106
wavelet-LHH_glszm_SizeZoneNonUniformity	0.0103
wavelet-HLH_glszm_LowGrayLevelZoneEmphasis	-0.0091
wavelet-HHH_glcm_Imc2	-0.0077
wavelet-LLH_glcm_Imc1	-0.0072
wavelet-HHH_glszm_SmallAreaLowGrayLevelEmphasis	-0.0066
wavelet-LHH_glrlm_GrayLevelNonUniformityNormalized	-0.0063
wavelet-LHH_glrlm_HighGrayLevelRunEmphasis	0.0059
wavelet-HHL_glszm_LargeAreaEmphasis	-0.0023
wavelet-LHH_glcm_Imc2	0.0022
wavelet-LLH_glrlm_ShortRunLowGrayLevelEmphasis	-0.0002

2）107 Peritumoral radiomics features and coefficients

feature	coef
wavelet-HHL_glcm_DifferenceEntropy	0.3749
wavelet-LLL_glcm_Id	-0.3385
original_shape_Sphericity	-0.3254
wavelet-HLH_glszm_GrayLevelNonUniformity	0.3043
wavelet-LHH_glcm_JointEntropy	-0.2737
original_glcm_DifferenceEntropy	0.2734
wavelet-LLL_glcm_Idmn	-0.2681
wavelet-HHH_glrlm_RunVariance	-0.2473
wavelet-HLL_glszm_ZoneVariance	0.235
wavelet-LHH_glszm_LargeAreaLowGrayLevelEmphasis	-0.2251
wavelet-LLH_gldm_DependenceNonUniformityNormalized	-0.2232
original_glcm_DifferenceAverage	0.2221
original_glcm_Correlation	0.2158
original_firstorder_Energy	-0.1843
wavelet-LHH_glcm_DifferenceVariance	0.181
wavelet-HLL_glszm_LowGrayLevelZoneEmphasis	-0.1696
wavelet-LHH_glszm_SmallAreaHighGrayLevelEmphasis	-0.1655
wavelet-LHL_glcm_ClusterProminence	-0.158
wavelet-HHH_glcm_SumEntropy	-0.1525
wavelet-HHH_glrlm_HighGrayLevelRunEmphasis	0.149
original_glcm_Id	-0.1425
wavelet-LLH_glrlm_RunLengthNonUniformityNormalized	0.1372

wavelet-HLL_glrlm_RunEntropy	-0.1284
wavelet-LHH_glszm_GrayLevelNonUniformityNormalized	-0.1274
wavelet-HLH_glszm_ZoneEntropy	0.1265
wavelet-HLL_firstorder_Skewness	-0.1251
wavelet-LLH_glszm_GrayLevelNonUniformity	-0.1246
wavelet-LHH_glszm_ZoneEntropy	0.123
wavelet-LLL_glcm_MaximumProbability	0.1198
wavelet-HLH_glszm_SmallAreaEmphasis	-0.1179
wavelet-HHH_firstorder_Minimum	0.1141
wavelet-HHH_gldm_DependenceEntropy	0.1136
original_shape_Elongation	-0.1134
wavelet-HHL_glcm_Correlation	-0.1132
wavelet-HLL_firstorder_Kurtosis	0.1083
wavelet-HHL_glcm_ClusterProminence	-0.1074
wavelet-LLL_gldm_LargeDependenceLowGrayLevelEmphasis	-0.0926
wavelet-LLH_glrlm_RunVariance	0.0817
wavelet-HHH_firstorder_Skewness	-0.0793
wavelet-LLL_firstorder_Kurtosis	0.0791
original_glszm_LowGrayLevelZoneEmphasis	0.077
wavelet-LLH_glcm_Idmn	0.0768
wavelet-HLL_glcm_Contrast	0.0751
wavelet-LLH_glcm_ClusterProminence	0.0745
wavelet-HLH_glrlm_ShortRunLowGrayLevelEmphasis	-0.0715
wavelet-HLH_glcm_SumEntropy	-0.0711
original_firstorder_Maximum	-0.071
wavelet-HLH_glszm_GrayLevelNonUniformityNormalized	0.0702
original_firstorder_Minimum	-0.0699
wavelet-HLH_glrlm_ShortRunHighGrayLevelEmphasis	-0.0696
wavelet-HHH_glszm_ZoneEntropy	0.0674
wavelet-LHL_glcm_ClusterShade	0.0673
wavelet-HHL_firstorder_Median	-0.064
wavelet-HLL_glcm_Id	-0.0638
wavelet-HHH_glcm_Autocorrelation	-0.0637
wavelet-HHH_glcm_SumSquares	0.0626
wavelet-LHL_glcm_Correlation	-0.0579
wavelet-HHH_glszm_LargeAreaLowGrayLevelEmphasis	-0.0556
wavelet-LHL_firstorder_Skewness	0.0537
wavelet-HHL_glcm_MaximumProbability	-0.0527
wavelet-HHH_glrlm_GrayLevelVariance	0.05
wavelet-HLH_glszm_ZoneVariance	-0.05

wavelet-LHH_glcm_ClusterTendency	-0.0482
wavelet-HHL_glszm_LowGrayLevelZoneEmphasis	-0.0455
wavelet-LHH_gldm_LargeDependenceLowGrayLevelEmphasis	0.0441
wavelet-HLL_firstorder_RootMeanSquared	0.0421
wavelet-HLL_glcm_JointAverage	-0.0417
wavelet-HHH_firstorder_Kurtosis	0.0398
wavelet-LHL_glcm_MaximumProbability	-0.0398
wavelet-LHH_glrlm_LongRunLowGrayLevelEmphasis	-0.039
wavelet-LHH_firstorder_Range	0.0382
wavelet-HHL_glszm_SmallAreaEmphasis	0.038
wavelet-HHL_firstorder_Kurtosis	-0.0368
wavelet-HLH_firstorder_Skewness	0.0329
wavelet-HLH_firstorder_10Percentile	0.0303
wavelet-LHL_firstorder_Mean	0.0292
wavelet-HHL_glcm_Imc1	0.0284
wavelet-LHL_glcm_DifferenceAverage	0.0279
wavelet-HLH_firstorder_90Percentile	-0.0276
original_glszm_GrayLevelNonUniformity	0.0275
wavelet-LLH_firstorder_RootMeanSquared	-0.0218
original_firstorder_Skewness	0.0216
wavelet-HLH_glcm_ClusterTendency	-0.0213
wavelet-LLL_glrlm_ShortRunEmphasis	-0.0187
wavelet-HHL_glcm_Idmn	0.0177
wavelet-LHL_glszm_LowGrayLevelZoneEmphasis	0.0172
original_glrlm_LongRunLowGrayLevelEmphasis	0.0171
wavelet-HHH_firstorder_Mean	-0.017
wavelet-HLH_glszm_HighGrayLevelZoneEmphasis	0.0157
wavelet-LLH_firstorder_Skewness	0.0139
wavelet-LLH_glrlm_LongRunHighGrayLevelEmphasis	0.0138
wavelet-LHH_firstorder_Kurtosis	0.0135
wavelet-HLH_glrlm_GrayLevelVariance	-0.0135
original_glcm_Imc1	0.0119
wavelet-HHH_glszm_SmallAreaHighGrayLevelEmphasis	0.0118
wavelet-LHL_glrlm_RunEntropy	0.0098
wavelet-HLL_glcm_Correlation	-0.0097
wavelet-LLH_glszm_LargeAreaEmphasis	-0.0085
wavelet-HHH_firstorder_10Percentile	0.0078
wavelet-HHH_glszm_LowGrayLevelZoneEmphasis	-0.0071
wavelet-LLH_firstorder_Range	-0.0069
wavelet-HHL_glcm_ClusterShade	-0.0065

wavelet-LHH_firstorder_90Percentile	-0.0044
wavelet-HLH_glrlm_LongRunLowGrayLevelEmphasis	0.0029
wavelet-HHH_glszm_SizeZoneNonUniformityNormalized	-0.0028
wavelet-HLL_glcm_ClusterProminence	-0.0017
wavelet-LHH_firstorder_Skewness	-0.0004

3）130 Combined radiomics features and coefficients

feature	coef
wavelet-LLL_glcm_DifferenceEntropy	1.398
wavelet-LHH_glrlm_RunLengthNonUniformityNormalized	-0.8616
original_glcm_Imc1	0.8168
wavelet-LHH_glrlm_RunEntropy	-0.5484
slice_index	-0.4674
wavelet-HHL_glcm_Id	-0.4672
wavelet-LHH_glszm_ZoneEntropy	0.4223
wavelet-LLH_firstorder_Variance	0.3548
wavelet-LHH_glcm_SumEntropy	-0.3362
original_glcm_Id	-0.3136
wavelet-LHH_glszm_SizeZoneNonUniformityNormalized	0.3124
wavelet-LLH_firstorder_Range	-0.303
wavelet-HLL_gldm_DependenceEntropy	-0.293
wavelet-LLH_glcm_SumEntropy	0.2906
wavelet-LLL_firstorder_MeanAbsoluteDeviation	0.2793
wavelet-HHH_glrlm_LongRunEmphasis	-0.2729
wavelet-HLL_glszm_LowGrayLevelZoneEmphasis	-0.2673
wavelet-HHH_firstorder_Kurtosis	0.2527
wavelet-HLH_glszm_GrayLevelNonUniformity	0.25
wavelet-LHH_gldm_LargeDependenceHighGrayLevelEmphasis	-0.2492
wavelet-HLL_firstorder_RootMeanSquared	0.2452
wavelet-HHH_firstorder_Range	-0.2445
wavelet-LHL_glcm_Correlation	-0.2439
wavelet-LLH_firstorder_Skewness	0.241
wavelet-HLH_glcm_MaximumProbability	0.2401
wavelet-HHH_glcm_ClusterProminence	0.2195
wavelet-HHH_glszm_SizeZoneNonUniformity	-0.2104
wavelet-LLH_gldm_SmallDependenceLowGrayLevelEmphasis	-0.2086
wavelet-HHL_firstorder_90Percentile	0.205
wavelet-LLH_glszm_SizeZoneNonUniformityNormalized	0.2002
wavelet-HLH_glcm_Autocorrelation	-0.194
original_firstorder_Maximum	-0.1897

wavelet-HHH_glszm_SizeZoneNonUniformityNormalized	0.1883
wavelet-HLH_firstorder_Range	-0.1853
wavelet-HHH_glcm_SumEntropy	-0.1742
wavelet-LHL_firstorder_Mean	0.1697
wavelet-HLH_glrlm_HighGrayLevelRunEmphasis	0.1458
wavelet-LLL_glcm_JointAverage	-0.1452
wavelet-LHL_firstorder_Range	-0.1447
wavelet-HHL_firstorder_Maximum	-0.1413
wavelet-HLH_glcm_SumEntropy	-0.1398
wavelet-HHL_glcm_ClusterShade	0.1398
wavelet-LHH_glcm_Imc2	-0.1366
wavelet-HLL_firstorder_Median	0.1343
wavelet-HLH_firstorder_Kurtosis	0.1335
wavelet-HLL_glcm_JointAverage	-0.1282
wavelet-HLH_glrlm_LongRunEmphasis	0.1282
wavelet-LLL_firstorder_Kurtosis	0.1279
wavelet-LHL_glcm_MaximumProbability	-0.1251
wavelet-LHH_gldm_LargeDependenceLowGrayLevelEmphasis	-0.1245
wavelet-HLL_glcm_Correlation	-0.1238
wavelet-HLL_glszm_LargeAreaEmphasis	0.1235
wavelet-LHL_glcm_Id	0.1227
wavelet-HLH_glcm_Imc1	0.121
wavelet-LHH_glszm_SizeZoneNonUniformity	-0.121
wavelet-LHL_glcm_ClusterProminence	-0.1205
wavelet-LHL_firstorder_Kurtosis	0.1141
wavelet-HLH_glrlm_RunLengthNonUniformityNormalized	-0.1125
wavelet-LHH_gldm_DependenceVariance	0.1111
wavelet-HHH_glrlm_GrayLevelNonUniformityNormalized	-0.1107
wavelet-LLL_glcm_DifferenceVariance	-0.1103
wavelet-HHH_gldm_DependenceVariance	0.1066
wavelet-HHH_glszm_SmallAreaHighGrayLevelEmphasis	0.1042
wavelet-HLH_firstorder_Skewness	-0.1035
wavelet-LLH_firstorder_RootMeanSquared	0.1009
wavelet-LLH_glrlm_ShortRunHighGrayLevelEmphasis	-0.1001
wavelet-HHH_glszm_LargeAreaLowGrayLevelEmphasis	0.0989
wavelet-LLH_firstorder_10Percentile	-0.0922
wavelet-HLH_glszm_SizeZoneNonUniformityNormalized	0.0917
wavelet-HHH_glszm_GrayLevelNonUniformityNormalized	0.0915
wavelet-LHH_glrlm_RunVariance	0.0875
wavelet-LLL_glszm_LargeAreaLowGrayLevelEmphasis	-0.0838

original_glcm_Correlation	0.0815
wavelet-HLH_glszm_SmallAreaLowGrayLevelEmphasis	-0.08
wavelet-HLH_firstorder_Uniformity	-0.0773
wavelet-HHL_firstorder_Median	0.0753
wavelet-LLH_glcm_Imc1	-0.074
wavelet-LHH_glszm_SmallAreaHighGrayLevelEmphasis	-0.0717
wavelet-HLH_glrlm_GrayLevelVariance	-0.0682
wavelet-HHH_glrlm_ShortRunHighGrayLevelEmphasis	0.0682
wavelet-HLL_glcm_Idmn	-0.0679
wavelet-HHH_firstorder_Mean	0.066
wavelet-LHH_firstorder_Kurtosis	-0.0658
wavelet-HLH_gldm_LargeDependenceHighGrayLevelEmphasis	-0.062
wavelet-HHH_firstorder_Median	-0.0596
wavelet-HHH_glcm_MaximumProbability	-0.0594
original_shape_Elongation	0.0579
wavelet-LLL_glcm_Imc2	0.0563
wavelet-HHL_glcm_Correlation	-0.0547
wavelet-LHH_glszm_GrayLevelNonUniformityNormalized	0.0541
wavelet-HHH_glszm_GrayLevelNonUniformity	0.053
wavelet-LHL_glcm_JointAverage	-0.0526
wavelet-HLH_gldm_DependenceVariance	-0.0518
wavelet-HLH_glszm_SmallAreaHighGrayLevelEmphasis	-0.0482
wavelet-HHH_firstorder_Minimum	0.0477
wavelet-HHL_glszm_LargeAreaEmphasis	-0.046
wavelet-HHH_gldm_LargeDependenceLowGrayLevelEmphasis	-0.0446
wavelet-LHH_glrlm_GrayLevelVariance	0.0441
wavelet-HHH_glrlm_HighGrayLevelRunEmphasis	0.0439
wavelet-LHH_firstorder_Maximum	0.0429
wavelet-HLH_firstorder_90Percentile	0.0406
wavelet-LHH_firstorder_Mean	-0.0404
wavelet-HHL_glcm_Imc2	-0.0365
wavelet-HLH_glszm_GrayLevelVariance	0.0354
wavelet-HLH_glrlm_ShortRunLowGrayLevelEmphasis	-0.0347
wavelet-HHH_glszm_ZoneEntropy	0.0336
wavelet-HHH_glszm_SmallAreaLowGrayLevelEmphasis	0.0327
wavelet-HHL_firstorder_Mean	0.0321
wavelet-HHL_glcm_ClusterProminence	0.03
wavelet-LLH_glrlm_LongRunLowGrayLevelEmphasis	-0.0295
wavelet-HLH_firstorder_Minimum	0.0291
wavelet-HLH_glszm_LowGrayLevelZoneEmphasis	0.0291

wavelet-LHH_glszm_SmallAreaLowGrayLevelEmphasis	0.0286
original_glcm_Imc2	-0.0285
wavelet-HHL_glcm_Idmn	0.0257
wavelet-HHH_glszm_LowGrayLevelZoneEmphasis	0.0188
wavelet-HHH_glrlm_ShortRunLowGrayLevelEmphasis	0.0185
wavelet-LHH_glrlm_ShortRunLowGrayLevelEmphasis	0.0177
wavelet-LLH_glszm_HighGrayLevelZoneEmphasis	-0.0152
wavelet-HLL_glcm_Id	-0.0134
original_firstorder_Skewness	-0.0132
wavelet-LHH_glszm_GrayLevelNonUniformity	-0.0128
wavelet-HHH_glcm_SumSquares	0.0101
wavelet-LHH_glrlm_ShortRunHighGrayLevelEmphasis	0.0079
wavelet-LHH_glszm_LowGrayLevelZoneEmphasis	-0.0071
wavelet-HHL_gldm_DependenceEntropy	0.0058
original_glcm_ClusterShade	-0.0035
wavelet-LHL_glcm_ClusterShade	0.0026
wavelet-LHL_gldm_DependenceEntropy	0.0018
wavelet-LHL_firstorder_Median	-0.0003
